# News and Gossip

**Published:** 1868-07-15

**Authors:** 


***News and Gossip.***

The most astonishing case of monomania on record is the adhesion of Corydon L. Ford to the dilapidated fortunes of A. B. P. *Can* a man lie down with dogs and not get up covered with fleas?——Robert C. Kedzie, M.D., of Lansing, Mich., has been appointed to the vacancy occasioned by the resignation of Prof. Armor, in the University of Michigan. From our knowledge of Dr. Kedzie, we do not believe he will accept the position. If he does, we can only exclaim: *Quantum mutatus ab illo!*——Some gentleman from Detroit, whose name we can not recall, has been appointed to the chair of Surgery. G. D. Beebee, of this city, was overslaughed. ——A. B. P. wants to get a professorship in Brooklyn, but the island gentlemen "can't see it." C. L. F. is adhering to him like a blister, to *draw* him there. P. thinks the Flints and Hamilton are "jealous of him!" Another instance of the fly on the bull's horn.——The Detroit College hangs fire as yet. But the gentlemen who have organized it are plucky, and understand "the situation." State institutions are always the tools of political hacks. Independence is the guarantee of success. The Michigan school proves the old adage: "Money makes the mare go;" but as she had in this case beggars on her back, of course she went to the devil. Good bye, D—s! ——They use *Carbol. acid* in Bellevue as a dressing, etc., in the proportion of 5 parts of *Linseed oil* to 1 part *Carbol. acid.* Or, as a wash, in the proportion of about 5 or 6 grs. to ℥i. water.

Our friend, Dr. R. S. Kelso, of Blooming Grove, Ill., in a private note, details the following interesting case of

